# Preoperative MRI is helpful but not sufficient to detect associated lesions in patients with chronic ankle instability

**DOI:** 10.1007/s00167-017-4567-x

**Published:** 2017-05-15

**Authors:** Kevin Staats, Manuel Sabeti-Aschraf, Sebastian Apprich, Hannes Platzgummer, Stephan E. Puchner, Johannes Holinka, Reinhard Windhager, Reinhard Schuh

**Affiliations:** 1 Department of Orthopaedic Surgery, Medical University of Vienna, Waehringer Guertel 18-20, 1090 Vienna, Austria; 2 Department of Biomedical Imaging and Image-guided Therapy, Medical University of Vienna, Waehringer Guertel 18-20, 1090 Vienna, Austria

**Keywords:** Ankle, Instability, Arthroscopy, MRI

## Abstract

**Purpose:**

The aim of this study was to determine the reliability and validity of preoperative magnetic resonance imaging (MRI) scans for the detection of additional pathologies in patients with chronic ankle instability (CAI) compared to arthroscopic findings.

**Methods:**

Preoperative MRI images of 30 patients were evaluated regarding articular and periarticular comorbidities and compared to intraoperative findings. The reliability of MRI was determined by calculating specificity, sensitivity, as well as positive and negative predictive values. The accuracy of the classification of cartilage lesions by Outerbridge and Berndt and Harty rating scales was determined by calculating the area under the receiver operating curve (AUC).

**Results:**

In total, 72 additional pathologies were found arthroscopically compared to 73 lesions gathered from MRI images. Sensitivity ranged from 89% for peroneal tendinopathy to 28% for additional ligamentous lesions. Specificity ranged from 100% for anterolateral impingement, loose bodies and peroneal tendinopathy to 38% for additional ligamentous lesions. For cartilage lesions, sensitivity was at 91% and specificity was at 55% for the Outerbridge grading scale. For the Berndt and Harty classification system, sensitivity was at 91% and specificity was at 28%. Correlation of additional pathologies ranged from weak (*r*_s_ = 0.48; *p* = 0.02) to moderate results (*r*_s_ = 0.67; *p* < 0.001).

**Conclusion:**

CAI is associated with a high incidence of additional pathologies. In some cases, MRI delivers insufficient results, which may lead to misinterpretation of present comorbidities. MRI is a helpful tool for preoperative evaluation, but arthroscopy remains gold standard in the diagnosis of associated lesions in patients with CAI.

**Level of evidence:**

III.

## Introduction

Lateral ankle sprains are one of the most common injuries caused by stressful inversion [8, 24, 26]. Despite the fact that most patients can be treated conservatively with success, approximately 30% of the patients with ankle sprains remain with chronic lateral ankle instability (CAI) [12]. With recurrent sprains and persistent pain, a subset of CAI patients requires surgical treatment due to a mechanical dysfunction of the lateral ligamentous complex [3, 15]. Surgical treatment aims for anatomical repair [24]. This can be achieved arthroscopically or through an open approach by transosseous fixation of the torn ligamentous fibres to the anteroinferior aspect of the fibula [20]. Additionally, the inferior extensor retinaculum can be augmented to the fibular periosteum [5].

Isolated injuries of the lateral ligamentous complex are scarce and additional lesions are commonly investigated in CAI [16]. In many cases, this is due to pathomechanical forces acting on the unstable ankle [18]. Peroneal tendon pathologies, concomitant ligamentous and syndesmotic injuries, anterolateral impingement or osteochondral lesions are the most common associated injuries [8, 23]. Although magnetic resonance imaging (MRI) represents a standard diagnostic procedure, information about reliability and validity remains inconclusive [4, 21, 27]. Therefore, it is still debated whether MRI delivers exact and reliable preoperative information about the extent involved injured structures [14]. Due to the advantage of direct visualization of intraarticular structures, arthroscopy is considered as gold standard [4, 10, 11, 21, 25].

The aim of this study was to determine the reliability and validity of preoperative MRI scans for the detection of additional bony, ligamentous, musculous and soft tissue-related pathologies in patients with CAI compared to arthroscopic findings. We hypothesized that compared to arthroscopic findings, preoperative MRI may lead to misinterpretation of the extent and the amount of additional pathologies in patients with CAI. Literature about the evaluation of additional pathologies in CAI is scarce and most authors only focused on certain pathologies. This present study should give the reader an insight on the reliability of preoperative MRI of CAI for the most common additional pathologies combined.

## Materials and methods

Thirty patients with CAI and failed conservative treatment were included in this retrospective investigation. All patients presented with recurrent ankle sprains in their medical history. Physical examination was performed by two orthopaedic surgeons (RS and MS) with years of experience in foot and ankle surgery. CAI was suspected in patients with positive anterior drawer test and/or positive talar tilt test. CAI was subsequently diagnosed if MRI revealed rupture or partial rupture of the anterior talofibular ligament (ATFL). All patients received an anatomical reconstruction with suture anchor technique between February 2012 and January 2016. Table 1 summarizes the demographic data of all patients included.

**Table 1:** Demographic data of all patients involved

Variable	Data
Age, years	39.1 ± 15.1 (18–71)
Sex	Male *n* = 15/female *n* = 15
Side involved	Left *n* = 14/right *n* = 16
Symptom duration, weeks	28.4 ± 12.7 (13–49)
Time between MRI and arthroscopy, months	3.9 ± 3.5 (0–15)

Data are shown as mean with ± standard deviation and (range) unless otherwise indicated

### MR imaging and analysis

Preoperative MRI examination was performed using a 3 T scanner (Siemens Magnetom Trio, A Tim System; gradient strength of 40 mT/m) with a dedicated 8-channel foot/ankle coil. Patients were placed in feet-first supine position with the ankle joint in the centre of the scanner. A standard native morphological MRI protocol containing the following sequence was used: (1) An axial T2-weighted (T2-w) turbo spin echo (TSE) sequence with fat suppression (fs) (repetition time (TR) 5520 ms, echo time (TE) 118 ms, bandwidth (BW) 203, field of view (FoV) 150 × 150, matrix 346 × 364, slice thickness (SL) 3 mm, acquisition time (TA) 02:57 min) (2) a sagittal T1-w turbo inversion recovery magnitude (TIRM) sequence (inversion recovery 220 ms, TR 6000 ms, TE 51 ms, BW 252, FoV 180 × 180, matrix 256 × 320, SL 3 mm, TA 04:25 min), (3) a sagittal T1-w spin echo (SE) sequence (TR 548 ms, TE 16 ms, BW 203, FoV 180 × 180, matrix 288 × 384, SL 3 mm, TA 05:55 min), (4) an axial proton density-weighted (PD-w) spectral attenuated inversion recovery (SPAIR) sequence (TR 3500 ms, TE 31 ms, BW 191, FoV 180 × 180, matrix 240 × 320, SL 3 mm, TA 03:06 min) and (5) a coronal PD-w SPAIR sequence (TR 5100 ms, TE 48 ms, BW 352, FoV 200 × 200, matrix 308 × 384, SL 3 mm, TA 06:08 min). Total scan time including a set of localizers was approximately 24 min.

All images were reviewed independently by one orthopaedic resident (KS) with special interest in MRI and ankle surgery, including monitoring dissections of anatomic specimens and previous training in MRI of the ankle joint, and one reader with years of experience in foot and ankle surgery (RS) on a picture archiving and communication system (PACS) workstation. The readers were blinded to the patient’s medical history and arthroscopic findings. Ligamentous lesions were diagnosed using the following criteria described by Joshy et al. [14]: discontinuity, curved contour, non-visualization or signal enhancement within the ligament. Cartilage lesions found during arthroscopy were evaluated using the Outerbridge grading scale [22]. Arthroscopically observed cartilage lesions were compared to preoperative MRI images by using the classification system published by Berndt and Harty (B + H) and Outerbridge [2]. Additional comorbidities like anterolateral impingement, other ligamentous and syndesmotic pathologies, bony spurs, peroneal tendinopathy and loose bodies found in the MRI-based images were evaluated and detected using the criteria published by Linklater [19] and Alparslan et al. [1]. Hypertrophic synovium and fibrosis causing anterolateral impingement was characterized on MRI as an abnormal soft tissue mass in the anterolateral gutter showing low T1- and low to intermediate T2-signals [13].

### Surgical technique

All patients underwent anatomical lateral ligament reconstruction with suture anchor technique. A 2.7-mm arthroscope and standard anteromedial and anterolateral portals were used. Prior to the reconstructive procedure, a full diagnostic arthroscopy with adequate documentation was performed. Pathologies of the lateral ligament complex were confirmed under direct visualization with a forceful inversion manoeuvre. A ligamentous pathology was diagnosed when a non-anatomical/abnormal course of the ligament, a decreased tensity or discontinuity of the ligament were detectable. Bony spurs and other tissue causing potential impingement as well as potential loose bodies were removed extensively. For mini-open ligament repair resorbable suture anchors were used (BioMini-Suture-Tak^®^, Arthrex).

### Intraoperative evaluation

All arthroscopic findings were retrospectively reviewed by an experienced orthopaedic surgeon (RS) and a trained assessor (KS) independently. Again the readers were blinded to the patient’s name and medical history. Surgical records and intraoperative photographic documentation were examined considering ligamentous pathologies, osteochondral lesions by using the Outerbridge grading system, the presence of anterolateral impingement, loose bodies, bony spurs and peroneal tendinopathy.

This study was approved by the local ethics committee of the Medical University of Vienna (EK No. 1072/2016).

### Statistical evaluation

The frequency of pathologic findings from preoperative MRI scans and intraoperative documentation are compiled to calculate sensitivity, specificity, positive predictive value (PPV), negative predictive value (NPV) with lower and upper 95% confidence interval (CI) levels. Accuracy for the detection of cartilage damage with both classification systems (Outerbridge and B + H) is determined by calculating the area under the receiver operating curve (with 95% CI). For the evaluation of a potential correlation between radiologic and arthroscopic features, Spearman’s rank order correlation was used and expressed as *r* values (*r*_s_). Arthroscopic findings were accounted for standard of reference. Interrater and intrarater reliability was assessed by comparing the independent results of two evaluation cycles. The readers were able to reproduce their own findings 4 weeks apart. The agreement between the two readers was excellent to good, depending on the comorbidity and modality. Table 2 displays the inter- and intrarater reliability of the additional lesions found in MRI and arthroscopy. Basic descriptive statistics were performed using SPSS software version 23 (SPSS Inc., Chicago, USA), and *p* values < 0.05 were considered as statistical significant.

**Table 2:** Inter- and intrarater reliability of MRI and arthroscopic findings

	Interrater MRI	Interrater arthroscopy	Intrarater MRI	Intrarater arthroscopy
Anterolateral impingement	0.851	0.998	0.949	1
Anterior tibial bony spur	0.833	0.969	0.903	0.976
Peroneal tendinopathy	0.734	0.886	0.884	0.982
Loose bodies	0.737	0.987	0.992	1
Additional ligament lesions	0.702	0.823	0.792	1

Inter- and intrarater reliability was assessed by comparing the results of two independent readers two evaluation cycles 4 weeks apart

In addition, a post hoc sample size calculation indicated we had 0.81 power to detect an effect size of 0.47, with alpha set at 0.05. Sample size was calculated using free sample size calculating software G*Power version 3.1.9.2 (Franz, Universitat Kiel, Germany).

## Results

### Arthroscopic and MRI-based findings

All patients (*n* = 30) showed changes (total rupture: *n* = 16, partial rupture: *n* = 6, elongation: *n* = 8) both in MRI and arthroscopically in the course of the ATFL, with all of these requiring surgical treatment in terms of an anatomical ligament repair using suture anchors for refixation.

**Fig. 1:**
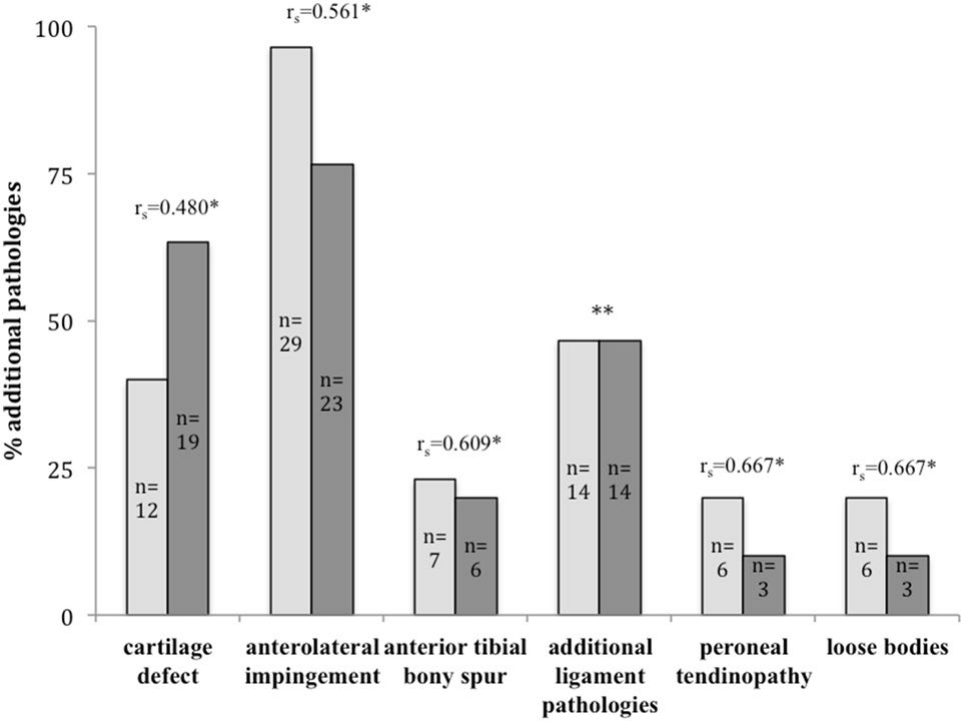
Prevalence and correlation of additional lesions in CAI patients. Lesions were detected intraoperatively (*bright grey*) and in MRI (*dark grey*). Correlation was determined by calculating Spearman’s rank coefficient (*r*_s_); (*statistically significant), **correlation not calculated because of misleading results due to different entities found in MRI and arthroscopy (Table 3)

Overall, 72 additional pathologies arthroscopically compared to 73 lesions in MRI images were found in this study. A per-patient analysis illustrated that each individual showed an average of two comorbidities intraoperatively (range 1–4) and radiologically (0–6). Figure 1 and Table 3 summarize all additional pathologies found during arthroscopy and in preoperative MRI scans.

**Table 3:** Reliability of MRI for additional pathologies

	Sensitivity	Specificity	PPV	NPV
Anterolateral impingement	79.3% (CI 60–91)	100% (CI 5–100)	100% (CI 82–100)	14.3% (CI 0–58)
Anterior tibial bony spurs	57.1% (CI 20–88)	91.3% (CI 70–98)	66.3% (CI 24–94)	87.5% (CI 67–97)
Peroneal tendinopathy	89.0% (CI 70–97)	100% (CI 31–100)	50.2% (CI 14–86)	100% (CI 83–100)
Loose bodies	33.3% (CI 9–69)	100% (CI 83–100)	100% (CI 31–100)	80.1% (CI 61–92)

Reliability was evaluated by calculating sensitivity, specificity, positive predictive value (*PPV*) and negative predictive value (*NPV*). All values are shown with 95% confidence intervals (*CI*)

In 43% (*n* = 13) of all patients, additional ligament pathologies were documented intraoperatively, whereas in 33% (*n* = 10) preoperative MRI revealed an additional ligament lesion. Details on additional ligament pathologies are shown in Table 4.

**Table 4:** Additional ligament lesions found in MRI and arthroscopy (ASC)

	MRI/ASC	Sensitivity	Specificity	PPV	NPV
CFL	4/5	80.0% (CI 30–99)	100% (CI 83–100)	100% (CI 40–100)	96.0% (CI 78–100)
PTFL	3/1	100% (CI 5–100)	86.7% (CI 76–99)	33.3% (CI 2–87)	100% (CI 84–100)
Deltoid lig.	5/0	–	83.3% (CI 65–94)	0% (CI 0–54)	100% (CI 83–100)
Basset lig.	1/8	0 (CI 0–40)	94.7% (CI 75–99)	0% (CI 0–95)	71.9% (CI 53–87)
Cervical lig.	1/0	–	96.2% (CI 81–99)	0% (CI 0–95)	100% (CI 85–100)
Total	14/14	28.4% (CI 10–58)	37.8% (CI 16–64)	29.3% (CI 10–58)	38.4% (CI 16–64)

Reliability of MRI is given by calculating specificity, sensitivity, positive predictive value (*PPV*), negative predictive value (*NPV*). All data are shown with 95% confidence interval (CI), *CFL* calcaneofibular ligament, *PTFL* posterior talofibular ligament*, lig. ligament*

### Correlation between preoperative MRI and arthroscopic findings

Correlation analysis for additional pathologies revealed low to moderate results with statistical significance (Fig. 1). An overall analysis of the numbers of pathologies evaluated for each patient from MRI and arthroscopy showed a weak but significant correlation (*r*_s_ = 0.41; *p* = 0.025).

Evaluation of cartilage damage in MRI and arthroscopy showed moderate results with high statistical significance for Outerbridge and poor to moderate correlation with statistical significance for B + H classification systems (Tables 5, 6).

### Sensitivity and specificity of preoperative MRI scans regarding additional cartilage injuries in CAI

**Fig. 2:**
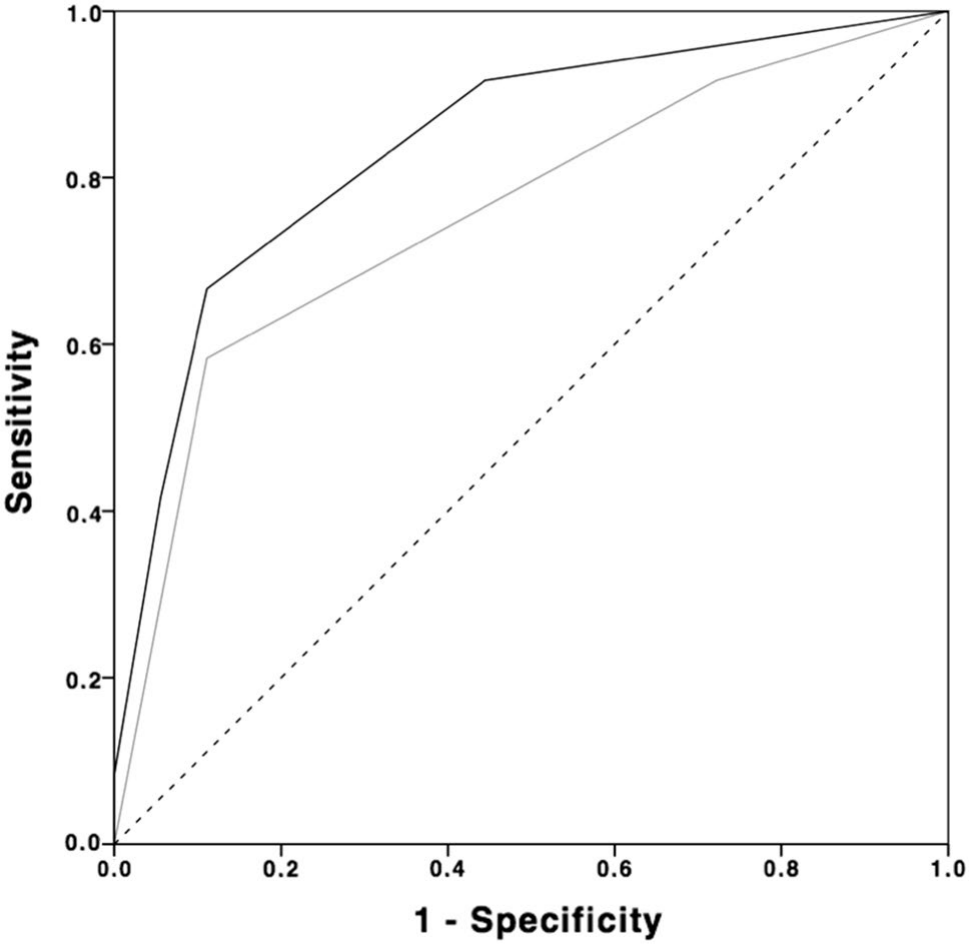
Accuracy of Outerbridge and Berndt and Harty classification in ankle MRI. Accuracy of Outerbridge grading scale (*black*) and Berndt and Harty (B + H) classification system (*grey*) was evaluated by calculating the area under the receiver operating curve (AUC). Accuracy for the Outerbridge classification system was 84% (*p* = 0.002; 95% CI range 69–99) compared to 76% (*p* = 0.02; 95% CI range 57–94) for B + H classification system

Table 6 gives a detailed summary of the results considering sensitivity and specificity. Accuracy for the Outerbridge classification system determined by the area under the receiver operating curve (AUC) was 84% (*p* = 0.002; 95% CI range 69–99) compared to 76% (*p* = 0.021; 95% CI range 57–94) for B + H classification system. Figure 2 shows the AUC for both classification systems.

**Table 5:** Incidence and classification of cartilage damage in MRI and arthroscopy

	Outerbridge MRI	Berndt + Harty MRI	Outerbridge arthroscopy
Grade I	47.4% (*n* = 9)	62.5% (*n* = 15)	8.3% (*n* = 1)
Grade II	21.1% (*n* = 4)	37.5% (*n* = 9)	8.3% (*n* = 1)
Grade III	26.3% (*n* = 5)		25.0% (*n* = 3)
Grade IV	5.3% (*n* = 1)		58.3% (*n* = 7)
TOTAL	19	24	12

Images were evaluated using the Outerbridge grading scale [22] (MRI and arthroscopy) and Berndt and Harty classification system [2] (MRI only)

## Discussion

The most important finding of this present study was that CAI was associated with a high incidence of additional pathologies in both MRI and arthroscopy, albeit with low sensitivity and high specificity, MRI may in some cases deliver underestimated information for present comorbidities. Therefore, standard morphological MRI may be a helpful tool for preoperative evaluation, but it cannot replace arthroscopy as gold standard for the diagnosis of comorbidities in CAI.

**Table 6:** Reliability/validity of MRI for cartilage defects

	Outerbridge classification	B + H classification
Sensitivity	91.7% (CI 61.5–99.7)	91.7% (CI 61.5–99.7)
Specificity	55.6% (CI 30.7–78.5)	27.8% (CI 9.7–53.5)
PPV	57.9% (CI 44.4–70.3)	45.8% (CI 37.7–54.2)
NPV	90.9% (CI 59.4–98.6)	83.3% (CI 39.9–97.4)
Correlation	*r*_s_ = 0.62; *p* < 0.001	*r*_s_ = 0.52; *p* = 0.003

Outerbridge grading scale and Berndt and Harty (B + H) classification system were applied. All values are shown with 95% confidence interval (CI). Correlation between MRI and arthroscopy was determined by calculating Spearman’s rank coefficient (*r*_s_)

*PPV* positive predictive value*; NPV* negative predictive value

MRI was most accurate for the detection of anterolateral impingement. These results are comparable to the findings from Clanton et al. for diffuse synovitis, albeit they could not detect any specificity for diffuse synovitis [7]. Even though we also gathered satisfying results for peroneal tendionpathy, we cannot approve this result unconditionally due to the low prevalence of peroneal lesions in our cohort. O’Neill et al. [21] conducted a study in which they also found that even though MRI delivers reliable results, the detection rate of additional lesions (chondral, loose bodies, peroneal tendon ruptures) in CAI patients ranged from 40 to 89%. Our data suggest that negative MRI results should be handled cautiously, especially regarding loose bodies. Only half of the loose bodies found intraoperatively were detected preoperatively through MRI. These results support the findings of O’Neill et al. [21] in which only 57% of loose bodies could be identified through MRI. In contrast, MRI showed a high sensitivity but a relatively low specificity and positive predictive values for cartilage defects. Gatlin et al. [9] found roughly similar results for preoperative MRI detecting cartilage defects of the talus in patients undergoing ankle surgery. Furthermore our results indicate that using the Outerbridge classification system seems slightly more accurate than using the classification system proposed by Berndt and Harty (Fig. 2). Superiority of the Outerbridge system may be caused by the possibility of applying this system intraoperatively by macroscopic examination of the cartilage.

With regard to a high incidence of comorbidities found in MRI and arthroscopically, our results confirm the findings of Choi et al. [6] and Kibler [17] in which they detected comorbidities in 96.9%, respectively, in 83% of observed patients who underwent lateral ligament reconstruction.

All observed pathologies showed only a low to moderate correlation between preoperative MRI and arthroscopy.

Arthroscopy fully agreed in four cases with additional ligament lesions only with the results of preoperative MRI. All of these patients showed lesions of the lateral ligament complex (CFL and/or PTFL). All other arthroscopically accounted ligamentous pathologies (*n* = 8) were a hypertrophic Basset ligament. MRI revealed five pathologic changes in the deltoid ligament, which could not be confirmed by the data gathered from arthroscopy. But since only patients with lateral ankle instability were included in this study there may have been some alterations in the ligaments of the medial complex that were not clinically present. Similar findings were reported by Cha et al., in which they also found low sensitivity for additional ligament pathologies [4].

This study represents several limitations:

First, retrospective analyses of arthroscopic images rely on the intraoperatively collected data and the experience of the reader. Both surgeons involved in the study are specialized in foot and ankle surgery. Although, a discrepancy in the experience between the readers was present (resident and experienced surgeon), a good to excellent inter- and intrarater reliability could be achieved. Therefore, this irregularity seems to be negligible. Second, due to the low number of patients, incidences may get over- or underestimated, and therefore, occurring differences between MRI and arthroscopy may appear more substantial. Third, the time between MRI and arthroscopy was prolonged in some cases. Thus, pathologies found in arthroscopy may not have been present during the time of MRI. But the mean time of 3 months from MRI to arthroscopy seems to be in an acceptable range.

## Conclusion

This study shows that CAI is associated with a high incidence of additional pathologies. For some comorbidities, MRI delivers low sensitivity and weak to moderate correlation to arthroscopic findings. Therefore, especially negative results in MRI should be handled with caution. MRI has shown to be a helpful tool for preoperative evaluation of additional lesions in patients with CAI. But diagnostic arthroscopy remains gold standard especially if a discrepancy between radiological and clinical findings is present.
